# Blood–brain barrier endothelial cells in neurodegenerative diseases: Signals from the “barrier”

**DOI:** 10.3389/fnins.2023.1047778

**Published:** 2023-02-24

**Authors:** Yiwen Yuan, Jian Sun, Qiang Dong, Mei Cui

**Affiliations:** ^1^Department of Neurology, Huashan Hospital, Fudan University, Shanghai, China; ^2^State Key Laboratory of Medical Neurobiology and Ministry of Education (MOE) Frontiers Center for Brain Science, Department of Neurology, Huashan Hospital, Fudan University, Shanghai, China

**Keywords:** blood–brain barrier, endothelial cells, neurovascular unit, neurodegenerative disease, Alzheimer’s disease, Parkinson’s disease

## Abstract

As blood–brain barrier (BBB) disruption emerges as a common problem in the early stages of neurodegenerative diseases, the crucial roles of barrier-type brain endothelial cells (BECs), the primary part of the BBB, have been reported in the pathophysiology of neurodegenerative diseases. The mechanisms of how early vascular dysfunction contributes to the progress of neurodegeneration are still unclear, and understanding BEC functions is a promising start. Our understanding of the BBB has gone through different stages, from a passive diffusion barrier to a mediator of central-peripheral interactions. BECs serve two seemingly paradoxical roles: as a barrier to protect the delicate brain from toxins and as an interface to constantly receive and release signals, thus maintaining and regulating the homeostasis of the brain. Most previous studies about neurodegenerative diseases focus on the loss of barrier functions, and far too little attention has been paid to the active regulations of BECs. In this review, we present the current evidence of BEC dysfunction in neurodegenerative diseases and explore how BEC signals participate in the pathogenesis of neurodegenerative diseases.

## 1. Introduction

Neurodegenerative diseases, such as Alzheimer’s disease (AD), Parkinson’s disease (PD), and amyotrophic lateral sclerosis (ALS), are defined as disorders characterized by progressive nervous system dysfunction (Ransohoff, 2016). Although the etiology and pathogenesis are still controversial and disputed subjects, most neurodegenerative disorders share many biochemical and neuropathological features. Nowadays, the blood–brain barrier (BBB) dysfunction has been identified as an early biomarker in neurodegenerative diseases since the strong evidence from neuroimaging, postmortem, and cerebrospinal fluid (Sweeney et al., 2018, 2019).

The BBB was originally regarded as a barrier for preventing blood cells, neurotoxic substances, and pathogens into the central nervous system (CNS). Specialized brain endothelial cells form the main part of the barrier and mediate the transport between cerebral capillaries and brain tissue (Zhao et al., 2015a). In contrast to those in other tissues, the membrane of the barrier-type brain endothelial cell (BEC) is continuous and sealed by extra intercellular junctional structures, such as tight junction protein zonula occludens-1 (ZO-1), claudins-1, -3, -5, and -12, and occludin. The unique structures of BECs result in fewer transcellular transports, low paracellular flow, and high transendothelial electrical resistance. However, increasing studies point out that the BBB is not just a static barrier but a dynamic and adaptable part of the neurovascular unit (NVU) (Neuwelt et al., 2011; Banks, 2019). According to the definition provided by Banks (2016), NVU is the BECs and the cells with which they interact, including astrocytes, neurons, microglia, mast cells, pericytes, and circulating immune cells. The maintenance of the adult BBB integrity and functions requires signaling from other cells in the NVU (Kaplan et al., 2020). Apart from transporting substances and information, the BEC itself is a source of informational molecules. These BECs constantly perform active work including maintaining the barrier functions and regulating the homeostasis of microenvironments in paracrine, autocrine, or endocrine ways, which has often been ignored (Ricard et al., 2021).

Barrier-type brain endothelial cell dysfunction or degeneration in BBB has been reported in neurodegenerative diseases for a long time (Grammas, 2011; Zlokovic, 2011; Di Marco et al., 2015; Sweeney et al., 2018). The discussion about the role of BECs tends to focus on the influx of circulating pathogens or neurotoxic materials through the broken BBB rather than the actions and conditions of BECs (Montagne et al., 2017; Sweeney et al., 2018). Additionally, recent advances in single-cell RNA sequencing revealed increasing evidence of BEC involvement in neurodegenerative diseases. This review aims to summarize the evidence of BEC dysfunction in neurodegenerative diseases and explore the signals of the BECs in the pathogenesis of neurodegenerative diseases. Last but not least, there are some brain endothelial cells which do not have barrier characteristics in the circumventricular organs, but this type of brain endothelial cells is outside the scope of this review. Therefore, BECs in this review represent the BECs.

## 2. Clinical and pathological evidence highlights BEC dysfunction in neurodegenerative diseases

### 2.1. Alzheimer’s disease

Alzheimer’s disease is a neurodegenerative disorder with prominent amnestic cognitive impairment. One of the pathology hallmarks of AD is the aggregation of pathological protein: β-amyloid (Aβ) extracellular plaques and tau neurofibrillary tangles. A classical hypothesis is the amyloid cascade hypothesis, in which the aggregation of Aβ precedes cortical tau pathology and contributes to neurodegeneration in AD (Hardy and Higgins, 1992).

Barrier-type brain endothelial cell dysfunction is considered to be one of the Aβ-induced pathological changes in AD (Thomas et al., 1996; Vagnucci and Li, 2003). For instance, increased remnants of capillaries, namely string vessels, are damaged vessels after BEC destruction, which have been reported in the AD brain for a long time (Brown, 2010).*In vitro*, different aggregation properties and heterogeneous compositions of Aβ peptides have distinct effects on endothelial cell viability, BBB integrity, and angiogenesis (Parodi-Rullan et al., 2020). Additionally, Aβ suppressed Wnt/β-catenin signaling by activation of GSK3β both *in vivo* and *in vitro* (Wang et al., 2022). The maintenance of BBB is dependent on the β-catenin signaling in BECs (Tran et al., 2016). Activation of the Wnt/β-catenin pathway upstream coreceptor the low-density lipoprotein receptor-related protein 6 (LRP6) in BECs effectively alleviates Aβ-induced pathologies in AD (Wang et al., 2022). Considering that dysregulation of the Wnt/β-catenin pathway has been reported in many neurodegenerative diseases, the LRP6 is a promising therapeutic target in other neurodegenerative diseases (Liu et al., 2014; Lengfeld et al., 2017; Lim et al., 2017).

On the other hand, damaged BECs accelerate the Aβ deposition and precede the progress of AD. Hippocampus neurons neighboring damaged BECs show strong oxidative DNA/RNA damage with increased Aβ precursor protein (APP) in AD patients (Sen and Hongpaisan, 2018). Based on these results, researchers suggest that Braak staging, the method to classify the degree of pathology in AD and PD, is supposed to refer to the levels of oxidative damage, the expression of APP or Aβ, and the number of total and degenerative BECs. Braak stages II–III can be characterized by strong oxidative damage with increased APP in neurons associated with an increased number of BECs, while Braak stages IV–VI may be characterized by an increased Aβ in neurons associated with increased numbers of total and degenerative BECs. However, BEC dysfunction is associated with neurodegeneration without affecting Aβ accumulation in another AD model, the aged tau-overexpressing mice (Tg4510 tauopathy model) (Bennett et al., 2018). The periods of obstructed blood flow in thin and spiraling vasculatures lead to cortical neurodegeneration in this model. The upregulation of numerous angiogenesis-related genes in BECs indicates that the BEC is the specific cell type driving these vascular changes in these aged tau mice (Bennett et al., 2018).

Indeed, the critical role of Aβ in AD pathogenesis has not escaped criticism for a long time. Panza et al. (2019) compared Aβ deposition to leukocytosis. As in sepsis, therapy is directed against the source of infection rather than the increasing white blood cells. Aβ accumulation might be a reaction of the brain to neuronal damage, and therapy should be targeted at the cause of neurodegeneration rather than the Aβ lesions. Zlokovic (2011) proposed the two-hit vascular hypothesis of AD to emphasize the role of vascular dysfunction in the initiation and progression of neurodegeneration diseases. In this hypothesis, vascular risks (hit one) cause BBB disruption and oligaemia, initiating the early steps in AD. The accumulation of Aβ is the result of BBB dysfunction (hit two), accelerating neurodegeneration and dementia (Zlokovic, 2011). Real data values for vascular dysfunction, Aβ deposition, metabolic dysfunction, functional impairment, and gray matter atrophy are integrated and analyzed with a multifactorial data-driven analysis (MFDDA) approach. The results point out that vascular dysregulation is the earliest and strongest pathologic biomarker in late-onset AD (Iturria-Medina et al., 2016). Mounting evidence continues to highlight that brain vasculature dysfunction happens before symptomatic onset and accelerates the severity of neurodegeneration during its early stages (Toledo et al., 2013; Vemuri et al., 2015; Arvanitakis et al., 2016; Malek et al., 2016; Nation et al., 2019).

Despite the finding that vascular dysfunction matters in the early stage of neurodegenerative diseases, more problems of early treatments are pressing for solutions, such as molecular mechanisms and promising drug targets. Recent advances in transcriptomics, particularly single-cell RNA sequencing, enable researchers to identify the key cells and signals in vascular pathology changes. In these discoveries, the vital roles of BECs in AD attract huge attention. So far, genome-wide association studies (GWAS) have uncovered plenty of disease-associated genomic loci in AD (Lambert et al., 2013; Jansen et al., 2019). BECs are enriched with the most AD GWAS genes in all kinds of cells, at least 30 of the top 45 AD GWAS genes in humans (Yang et al., 2022). Single-cell atlas from AD people display that upregulated genes in BECs are closely related to neurodegeneration, cytokine secretion, and immune responses (Grubman et al., 2019). To figure out the mechanisms of how genetic risk factors in BECs contribute to AD, more *in vitro* models have been used (Penney et al., 2020; Blanchard et al., 2022). Among these models, the human induced pluripotent stem cells (hiPSC) technique is the most mature and promising. The BECs from hiPSC with mutation of presenilin-1 (PSEN1) demonstrate alterations of tight junction proteins and efflux transporter expression, which suggests a BEC-targeted BBB disruption owing to familial AD PSEN1 mutation (Oikari et al., 2020). However, through single-cell RNA sequencing, bioinformatic analysis, and immunofluorescence, researchers reported that some hiPSC-derived BECs lack essential endothelial genes and transcription factors. From this perspective, despite the exciting breakthrough in the hiPSC technique and the promising applications in the workings of BEC dysfunction in AD, validation of hiPSC-derived BECs *in vitro* models should be highly regarded to reflect the pathological process accurately (Lu et al., 2021a,b).

### 2.2. Parkinson’s disease

Parkinson’s disease is the second most common neurodegenerative disorder with more than 6 million patients in the world. Bradykinesia, rest tremor, rigidity, and alterations in posture/gait are typical symptoms of PD. Loss of nigrostriatal dopamine neurons and the following imbalance between anti-kinetic and prokinetic activity contribute to these typical symptoms (Bloem et al., 2021). Vascular dysfunction in PD brain tissue is characterized by the clustering of BECs, vessel fragmentations, and the loss of capillary connections (Guan et al., 2013). String vessels, mentioned above in AD, are also significantly increased in PD patients, which suggests that BEC dysfunction is a common pathology in neurodegenerative diseases (Yang et al., 2015).

The aggregation of pathological protein α-synuclein in the form of intraneural Lewy bodies is the key pathological hallmark in PD. BEC alterations are observed mostly in the substantia nigra, locus coeruleus, and caudate putamen, where the aberrant α-synuclein aggregation is prominent. A 2.5-fold increase in the number of BECs is identified in the substantia nigra pars compacta of PD brain tissue, where the proangiogenic molecule vascular endothelial growth factors (VEGF) correspondingly upregulated because of the astrocytes activated by oligomeric α-synuclein (Faucheux et al., 1999; Wada et al., 2006). The newly created vessels not only appear in PD patients but also in the preclinical phase—incidental Lewy bodies disease, indicating an early involvement of BEC activation in the progress of PD (Desai Bradaric et al., 2012).

### 2.3. Amyotrophic lateral sclerosis

Amyotrophic lateral sclerosis, the third most common neurodegenerative disease, is characterized by the loss of motor neurons in the cortex, brainstem, and spinal anterior horn. Biomarkers for the diagnosis of ALS and the assessment of upper motor neuron abnormality in the early phase are limited, but neuroimaging techniques such as advanced magnetic resonance imaging (MRI) provide increasing insights into the pathophysiology of ALS (Wang et al., 2011). A correlation between ALS severity and gray matter perfusion suggests an early appearance of vascular dysfunction in ALS (Rule et al., 2010; Murphy et al., 2012). Capillary ultrastructural impairments and BBB disruption are also confirmed in post-mortem tissue from sporadic ALS patients (Sweeney et al., 2018). The overexpression of the P-glycoprotein transporter on BECs is associated with poor treatment efficiency in ALS (Mohamed et al., 2017).

### 2.4. Multiple sclerosis

Multiple sclerosis (MS) is an inflammatory-mediated demyelinating and neurodegenerative disease of the CNS. Many therapies for inflammation are available, while neurodegeneration and corresponding brain function impairments remain untargeted in MS. In progressive MS, the pathophysiological mechanisms of the demyelination and neurodegeneration are still unclear, which is important for new therapy and drugs development (Dangond et al., 2021).

In the pathogenesis of MS, peripheral immune cells cross the BBB into the brain and attack myelin membranes and neurons. BEC activation mediates leukocyte recruitment into CNS by the upregulation of adhesion molecules on them, which can be regarded as one of the biomarkers for the activity of MS (Cannella and Raine, 1995; Rossi et al., 2011). The expression of P-selectin on the BEC surface is significantly associated with disease activity in experimental models of MS (Fournier et al., 2017). Molecular MRI targeting at P-selectin is used for the detection of BEC activation, which is promising for clinical translation to monitor or even predict the activity and severity of MS, indicating BBB disruption and BEC activation in the early stage of MS (Gauberti et al., 2018). Moreover, recent single-cell transcriptomics identified that enhanced adhesive properties and immune transendothelial migration in MS were mainly localized in venous BECs (Fournier et al., 2023).

### 2.5. Huntington’s disease

Huntington’s disease (HD) is a monogenic neurodegenerative disorder characterized by a CAG repeat expansion in the Huntingtin (HTT) gene. In HD patients, similar BBB disruption and increase in blood vessel density have been identified in all major components of the NVU (Drouin-Ouellet et al., 2015). HiPSC-derived BECs from HD patients display intrinsic abnormalities in angiogenesis and barrier functions (Lim et al., 2017; Table 1).

**TABLE 1:** Clinical and pathological evidence highlights BEC dysfunction in neurodegenerative diseases.

Diseases	Alterations in BECs	Effects or meanings of BEC dysfunction	References
Alzheimer’s disease (AD)	Excessive production of neurotoxic substances from BECs with attendant alterations in BEC structure and function	Scavenging BEC-derived relaxing factors and producing potent oxidizing agents	Thomas et al., 1996
	BEC destruction and capillary remnants (string vessels)	Illustrating when and where there is vascular destruction at the capillary level in the process of AD	Brown, 2010
	High levels of RAGE and reduced LRP1 expression on BECs	Indicating that deficits in BEC-mediated Aβ clearance accelerate Aβ deposition and the following neurodegeneration	Donahue et al., 2006; Sagare et al., 2012; Wan et al., 2015; Storck et al., 2016
	High levels of oxidative damage and APP in neurons around BECs	Braak staging should refer to the levels of oxidative damage, the expression of APP or Aβ, and the number of total and degenerative BECs	Sen and Hongpaisan, 2018
	BEC-mediated pathological changes in blood vessels in tau-overexpressing mice (Tg4510)	BEC is the specific cell type driving tau-induced pathological changes in vasculature	Bennett et al., 2018
	Upregulation of genes related to neurodegeneration, cytokine secretion, and immune responses in BECs from AD patients	Providing insights into the contributions of BECs in AD	Grubman et al., 2019
	Enriched with 30 of the top 45 AD GWAS genes in human	Emphasizing the role of BECs in the pathophysiology of AD	Yang et al., 2022
Parkinson’s disease (PD)	Clustering of BECs, vessel fragmentations, and the loss of capillary connections	Vascular degeneration could be an additional contributing factor to the progression of PD	Guan et al., 2013
	BEC destruction and capillary remnants (string vessels)	Illustrating when and where there is vascular destruction at the capillary level in the process of PD	Yang et al., 2015
	A significant increase in the number of BECs in the brain tissues from PD and the preclinical phase of PD	Indicating an early involvement of BEC activation in the progress of PD	Faucheux et al., 1999; Wada et al., 2006; Desai Bradaric et al., 2012
Amyotrophic lateral sclerosis (ALS)	Upregulation of P-glycoprotein transporter on BECs	Associated with poor treatment efficiency in ALS	Mohamed et al., 2017
Multiple sclerosis (MS)	Upregulation of P-selectin on BECs	Associated with disease activity in experimental models of MS and can be used for the detection of BEC activation in clinic	Fournier et al., 2017; Gauberti et al., 2018
Huntington’s disease (HD)	Loss of angiogenesis and barrier functions in hiPSC-derived BECs	The Huntingtin gene induces BEC dysfunction and BBB disruption	Lim et al., 2017

BECs, barrier type brain endothelial cells; RAGE, receptor for advanced glycation end products; LRP1, low-density lipoprotein receptor-related protein 1; APP, Aβ precursor protein; GWAS, genome-wide association studies; Aβ, β-amyloid; hiPSC, human induced pluripotent stem cells.

## 3. BECs in the pathogenesis of neurodegenerative diseases

### 3.1. Dysregulation of BEC transports

Barrier-type brain endothelial cell transport system mediates the bidirectional trafficking of substances between blood and brain (Sweeney et al., 2019). The Aβ clearance mediated by BECs has been considered to be a key process in the pathogenesis of AD. Specific receptors on the BECs regulate the entry of plasma Aβ into the CNS and the clearance of brain Aβ. The receptor for advanced glycation end products (RAGE) mediates the entry of circulating Aβ into the brain, while the low-density lipoprotein receptor-related protein 1 (LRP1) is involved in transporting the Aβ out of the brain (Shibata et al., 2000; Deane et al., 2003). AD patients develop high levels of RAGE and reduced LRP1 expression on BECs (Donahue et al., 2006; Sagare et al., 2012). Aβ42 oligomer induces the upregulation of RAGE on BECs and BBB leakage *in vitro* (Wan et al., 2015). A multimodel RAGE blocker effectively suppresses Aβ-induced pathologies in the AD mice model (Deane et al., 2012). However, the clinical trial of the RAGE inhibitor did not show significant therapeutic effects (Galasko et al., 2014).

Endothelial-specific LRP1 knockout mice display reduced Aβ efflux from the brain and Aβ-dependent cognitive deficits (Storck et al., 2016). The LRP1-mediated Aβ transcytosis across BECs is regulated by PICALM, the phosphatidylinositol binding clathrin assembly protein (Zhao et al., 2015b). The PICALM/clathrin-dependent endocytosis mediates the internalization of the Aβ-LRP1 complex in BECs. In BECs, PICALM guides Aβ trafficking to Rab5-positive early endosomes and Rab11 that leads to Aβ transcytosis rather than Rab7, a GTPase that directs to lysosomes leading to degradation of ligands (Zhao et al., 2015b). Remarkably, the latest study reveals that artesunate, an FDA-approved anti-malaria drug, prevents the development of Aβ pathology in mice by elevating PICALM expression in BECs, suggesting a promising translation to human AD (Kisler et al., 2023). Likewise, maraviroc, a CCR5 antagonist, significantly reduces the HIV-induced AD-like brain pathologies, including Aβ deposition and tau hyperphosphorylation, which may result from the increased transendothelial Aβ transport via LRP1 pathways (Bhargavan et al., 2021). Interestingly, a recent study shows that loss of BEC LRP1 directly causes BBB breakdown, contributing to neurodegeneration and cognitive decline in an Aβ-independent way by activation of cyclophilin A-MMP9 pathway in BECs (Nikolakopoulou et al., 2021). This finding raises concerns about whether Aβ deposition is the initial factor in AD or just the reaction to BBB breakdown and endothelial deficits in Aβ clearance, but it still points out that increasing the LRP1 expression in BECs is beneficial in neurodegenerative diseases.

Apart from the Aβ transport across BECs, recent studies have paid attention to the exchange of α-synuclein, the pathological protein of PD, between the brain and peripheral tissues. Likewise, α-synuclein can cross the BBB in both directions, and α-synuclein can inhibit Aβ efflux in an LRP1-dependent manner (Sui et al., 2014). However, the polarized α-synuclein trafficking across BECs in the luminal-abluminal direction is directed by Rab7/VPS35 trafficking pathway, which is different from the Rab11-directed Aβ transcytosis in BECs (Alam et al., 2022). In addition, systemic inflammation promotes the α-synuclein-containing red blood cell (RBC)-derived extracellular vesicles (EVs) across BBB (Matsumoto et al., 2017). Some studies reveal that EV-derived α-synuclein can induce oligomerization of proteins in recipient cells and promote the spread of protein aggregates, but whether these RBC-derived EVs can induce a similar response in BECs has not been investigated (Bogale et al., 2021).

**FIGURE 1:**
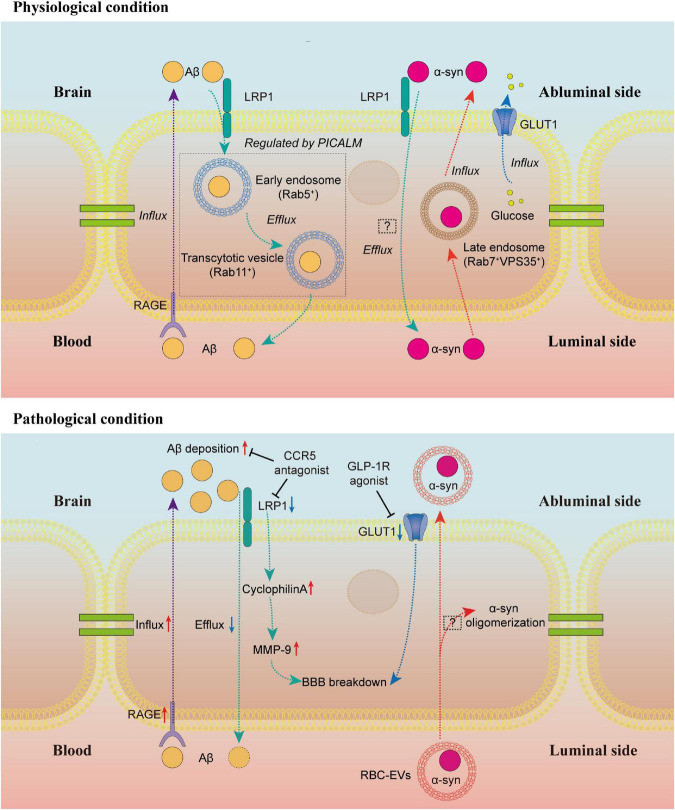
Brain endothelial cell transport in physiological and pathological conditions. Under physiological conditions, RAGE regulates the influx of Aβ to the brain, while LRP1 mediates the efflux of Aβ. The LRP1-dependent transendothelial Aβ transport is regulated by PICALM. PICALM guides Aβ to Rab5-positive early endosome and Rab11-positive transcytotic vesicle to the luminal side of BECs. The α-syn efflux is also an LRP1-dependent process, but the underlying mechanisms are still unclear. In the luminal-abluminal direction, the α-syn trafficking across BECs is directed by Rab7/VPS35 trafficking pathway. The GLUT1 is densely expressed on the abluminal membrane of the BECs, regulating the glucose transport across BBB. Under pathological conditions, the upregulation of RAGE and downregulation of LRP1 lead to the deposition of Aβ in the brain. The CCR5 antagonist is reported to alleviate the Aβ deposition by increasing the LRP1 expression. The LRP1 deficiency in BECs contributes to BBB breakdown in an Aβ-independent way by activation of cyclophilin A-MMP9 pathway in BECs. Similarly, the loss of GLUT1 in BECs leads to BBB breakdown. A GLP-1R agonist can reverse the reduced GLUT1 expression. The RBC-EVs can carry α-syn across BBB, but whether α-syn in these RBC-EVs can induce α-syn oligomerization in BECs like EV-derived α-syn in other recipient cells is still unclear. BEC, barrier type brain endothelial cell; RAGE, the receptor for advanced glycation end products; Aβ, β-amyloid; LRP1, the low-density lipoprotein receptor-related protein 1; PICALM, the phosphatidylinositol binding clathrin assembly protein; α-syn, α-synuclein; GLUT1, glucose transporter 1; BBB, blood–brain barrier; GLP-1R, glucagon-like peptide-1 receptor; RBC, red blood cell; EVs, extracellular vesicles.

Compared to other tissues, glucose transport across BECs needs to be tightly mediated to support the CNS. Disturbances of glucose uptake and metabolism in different brain areas are shown by ^18^F-fluoro-2-deoxyglucose positron emission tomography (FDG-PET) in neurodegenerative diseases (Chetelat et al., 2020; Ruppert et al., 2020). At a molecular level, glucose transporter 1 (GLUT1), the main glucose transporter across BBB, is densely expressed on the abluminal membrane of the BECs (Farrell and Pardridge, 1991). Lower GLUT1 levels are associated with BBB breakdown in AD patients (Kalaria and Harik, 1989). Specifically, the GLUT1 deficiency in BECs rather than astrocytes initiates the vascular phenotype with BBB breakdown and subsequent metabolic stress, contributing to early neurodegeneration in the AD mouse model (Winkler et al., 2015). Loss of BEC GLUT1 does not impair cerebral blood volume or BBB permeability but contributes to progressive neurodegeneration and CNS inflammation (Veys et al., 2020). The underlying mechanisms between reduced BEC GLUT1 and neurodegeneration remains unclear. Interestingly, a glucagon-like peptide-1 receptor (GLP-1R) agonist treatment significantly reversed multiply transcriptomic changes associated with AD in BECs, including reduced GLUT1 (Zhao et al., 2020). Although the exact pathways involved are still unclear, future research between the GLP-1R agonists and GLUT1 or other molecules in BECs may provide more therapeutic targets for early interventions of BBB breakdown in neurodegenerative diseases especially AD (Figure 1).

### 3.2. Neuroinflammation

Emerging evidence links neuroinflammation to neurodegenerative diseases (Heppner et al., 2015; Hammond et al., 2019; Mason and McGavern, 2022). Neuroinflammation concerning neurodegeneration is difficult to define because of the lack of specificity in AD, PD, and ALS. Among these diseases, neuroinflammation typically presents as reactive morphology of astrocytes and microglia, accompanied by some inflammatory mediators in the brain parenchyma (Ransohoff, 2016). Decades ago, simple *in vitro* studies confirmed that the neurotoxic factors from AD patients’ BECs caused neuronal death and promote the activation of microglia and astrocytes (Grammas et al., 1999; Grammas, 2011). The microglia, a type of tissue-resident macrophage in the brain, are recognized as the central cell in the neuroinflammation of neurodegenerative diseases (Hickman et al., 2018; Song and Colonna, 2018). Interestingly, recent studies have shown that some AD risk genes that mainly exist in the microglia of mice exhibit greater expression in human BECs, suggesting “a partial evolutionary transfer of AD risk genes and pathways from microglia to the vasculature from mice to humans” and a possible interaction between microglia and endothelial cells (Yang et al., 2022).

Owing to the specialized BECs, the healthy brain is an immune-privileged organ and poorly infiltrated by immune cells (Spadoni et al., 2017). Astrocyte-derived sonic hedgehog (SHH) promotes the immune quiescence of BECs by reducing their secretion of cytokines, chemokines and the expression of intercellular adhesion molecule-1 (ICAM-1) (Alvarez et al., 2011). In disease states, the endothelial cells undergo various changes, so-called endothelial cell activation (Hunt and Jurd, 1998). Activated BECs recruit immune cells and promote the trafficking of leukocytes across the BBB by secreting cytokines and colony-stimulating factors such as IL-1, IL-6, IL-8, and granulocyte-macrophage colony-stimulation factor (GM-CSF), while IL-25 released from BECs suppresses this process in the physiological condition (Sonobe et al., 2009). Under pathological conditions, leukocyte infiltration is a promising therapeutic target. Peripheral apolipoprotein E4 (ApoE4), a lipoprotein encoded by the strongest genetic risk factor for sporadic AD, is reported to upregulate multiple genes in BECs including leukocyte migration, immune processes, antigen processing, and presentation, as well as unfolded protein binding (Liu et al., 2022). In the MS mouse model, ApoER2 deficiency in BECs specifically reduces leukocyte rolling and endothelial adhesion, preventing paralysis and neuroinflammation (Calvier et al., 2021).

Activated BECs in aged mouse hippocampus express higher vascular cell adhesion molecule 1 (VCAM1), increasing microglial reactivity and impairing NPC activity (Yousef et al., 2019). Upregulation of VCAM1 is also reported in BECs activated by C3a/C3aR signaling, causing vascular dysfunction and age-associated neurodegeneration (Propson et al., 2021). Furthermore, microglia activation in the AD mouse model is reported to be C3aR-dependent, suggesting that C3a/C3aR/VCAM1 axis in BECs may play a crucial role in microglia activation of neurodegenerative diseases (Litvinchuk et al., 2018). Additionally, a recent study reported the soluble VCAM1 shed from VCAM1 activates microglia by binding the VLA-4 receptor and activating the p38 MAPK pathway in microglia, providing more details to this BECs signaling (Li et al., 2022).

Activated BECs can also recruit microglia and macrophages and induce a chronic proinflammatory condition in the perivascular area. A recent study has shown an unidentified role of BECs in the phagocytosis of myelin sheaths in the MS mouse model. The uptake of myelin debris activates BECs and promotes the secretion of pro-inflammatory mediators from them, causing the clustering of macrophages and microglia and other sequential events in the progression of MS (Zhou et al., 2019). Similarly, microglia clusters were found in the perivascular space in the PD mouse model (Elabi et al., 2021). Although the exact mechanisms of BEC-induced microglia cluster in neurodegenerative diseases remain mysterious, the chemokine CCL5 released by BECs is confirmed to attract the resident brain microglia during systemic inflammation. These vessel-associated microglia can maintain BBB integrity, while they may transform into phagocytic phenotype and impair BBB during sustained inflammation (Haruwaka et al., 2019). Whether the chemokine CCL5 leads to the clustered microglia in the perivascular area in neurodegenerative diseases needs to be verified by future research. Moreover, whether the protective effects of CCR5 antagonists in neurodegeneration mentioned above are associated with CCL5-induced microglia recruitment remains to explore. We hope that future research will tease out the mechanisms of the interactions of BECs and microglia in the early phase of neurodegenerative diseases, which may shed light on the neuroinflammation-induced BBB disruption and figure out the early intervention target.

Other than recruiting immune cells, BECs themselves modulate the immune response. Single-nucleus transcriptome analysis of prefrontal cortical samples from AD patients and healthy controls points out that some of the dysregulated pathways in BECs are related to antigen presentation (Lau et al., 2020). Interferon inflammation in BECs from APOE4 carriers is a promising target to interfere with cognitive decline in APOE4 carriers (Montagne et al., 2020; Yang et al., 2022). Unfortunately, these active roles of BECs in the immune response have not been deeply explored in the neuroinflammation of neurodegenerative diseases.

### 3.3. Regeneration failure

The occurrence of adult hippocampal neurogenesis in humans was first reported by Eriksson et al. (1998). The controversy about scientific evidence for this finding has raged unabated for 20 years (Kempermann et al., 2018). After the optimization of the human brain tissue processing methodologies, researchers not only provide evidence for the persistence of adult hippocampal neurogenesis both in physiological and pathological aging in humans but also reveal a progressively declined neurogenesis as AD advanced (Moreno-Jimenez et al., 2019). Terreros-Roncal et al. (2021) report common human adult hippocampal neurogenesis impairment in different types of neurodegenerative diseases. Although this conclusion has been criticized by several researchers, these technological, and conceptual advances provide some insight into the neurogenesis impairment in the pathogenesis of neurodegenerative diseases, which may be an opportunity to develop possible regenerative therapeutics (Alvarez-Buylla et al., 2022; Arellano et al., 2022).

Neurogenesis is defined as a systematic progress that neural stem cells (NSCs) generate new neurons. Adult neurogenesis takes place in the dentate gyrus (DG) hippocampus subfield and the subventricular zone (SVZ) in a continuous manner throughout aging. The extracellular microenvironment in DG and SVZ, namely the neurogenic niche, is a crucial regulator of stem cell behaviors *in vivo*. In these areas, BECs are in a specialized planar morphology to increase the contact area with the endfeet of adult NSCs (Palmer et al., 2000; Tavazoie et al., 2008; Kokovay et al., 2010; Boldrini et al., 2018). Secretions from BECs in the neurogenic niche regulate the proliferation, differentiation, and migration of NSCs (Shen et al., 2004, 2008; Bovetti et al., 2007; Whitman et al., 2009; Gomez-Gaviro et al., 2012). The contributions of BECs to neuronal migration and axon pathfinding have been well recognized (Peguera et al., 2021). Remarkably, BEC-dependent Dab1 signaling facilitates the communication between vessels and glia, which is necessary for the positioning of neurons during cortical development (Segarra et al., 2018). This outstanding research provides an important understanding of the crosstalk in the NVU and reveals the central role of BECs in neuro–glia–vessel communication.

Interestingly, there is a shared signaling “language” between NSCs and BECs. Some factors were originally identified by neurotrophic effects and later shown to regulate angiogenesis, and some angiogenesis factors were discovered to have effects on NSCs. A growing body of literature describes the neurotrophic effects of these factors in normal brain functions, which may be important targets in the pathogenesis of neurodegenerative diseases. For instance, a shared semaphorin pathway exists in neuronal and vascular morphogenesis (Gelfand et al., 2009). BEC-derived Sema3G is necessary for synaptic plasticity in the hippocampus. Specifically knockout of Sema3G in BECs impairs hippocampal synaptic structure, excitatory neurotransmission, and hippocampus-dependent memory in mice (Tan et al., 2019). Neurotrophins (NTs) are another important family of proteins mediating both neural and vascular functions (Chao, 2003; Lu et al., 2005). The brain-derived neurotrophic factor (BDNF) secreted mainly from BECs matters in the normal brain function such as cognition (Marie et al., 2018). BDNF reduces both in HD and AD patients. Although the exact brain area is different, increasing the level of BDNF is beneficial in these disease models (Blurton-Jones et al., 2009; Simmons et al., 2009). Additionally, the lack of VEGF contributes to neurodegeneration. Under physical conditions, the autocrine VEGF and TGFβ are crucial for the integrity of BBB (Ricard et al., 2021). The deletion of the hypoxia response element of VEGF establishes a typical ALS animal model with motor neuron degeneration (Oosthuyse et al., 2001). Moreover, lowering the level of VEGF in the familial ALS model accelerates the progress of ALS (Lambrechts et al., 2003). The protective effects of VEGF on motor neurons raise the potential for VEGF therapy (Keifer et al., 2014). Apart from ALS, preclinical studies present that delivery of VEGF to the brain is therapeutic in AD, PD, and HD (Yasuhara et al., 2005; Ellison et al., 2013; Herran et al., 2013; Religa et al., 2013; Garcia et al., 2014). However, recent studies have shown that higher level of VEGF demonstrates more pronounced BBB permeability and BEC dysfunction in AD, PD, and HD (Hsiao et al., 2015; Janelidze et al., 2015; Ali et al., 2022; Lan et al., 2022; Wood, 2022). These findings cause difficulties in the clinical applications of VEGF therapeutics in neurodegenerative diseases.

**FIGURE 2:**
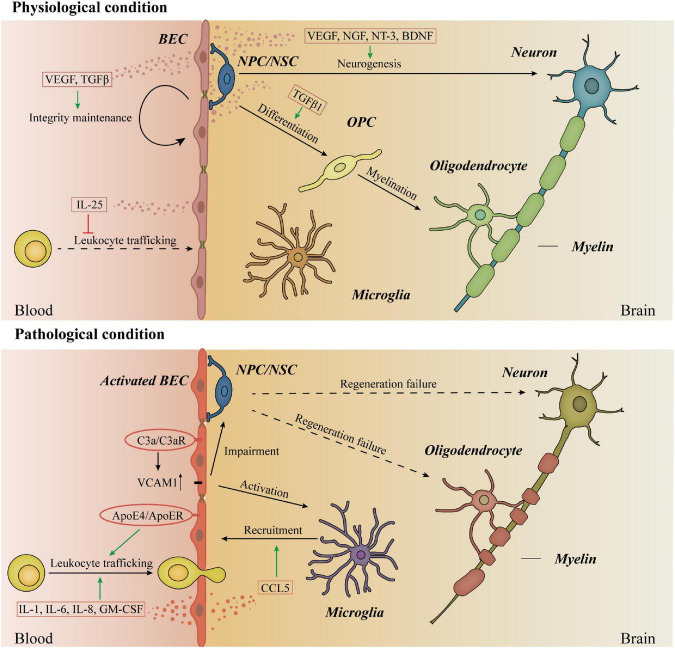
Immune responses and stem cell activities in physiological and pathological conditions. Under physiological conditions, BECs secret VEGF and TGFβ in the autocrine way to maintain the integrity of BBB. Moreover, IL-25 released from BECs inhibits leukocyte trafficking in physiological conditions. In the brain, BECs secret neurotrophic factors to regulate the proliferation, differentiation, and migration of NSCs, promoting neurogenesis. BEC-derived TGFβ1 regulates the differentiation of OPCs from NPCs. However, the exact role of BECs in the myelination process is still unknown. In disease states, activated BECs secret IL-1, IL-6, IL-8, and GM-CSF to promote the trafficking of leukocytes across BBB. Periparial ApoE4 activates BECs and promotes leukocyte trafficking across BBB. The upregulation of VCAM1 in BECs is regulated by the binding of C3a and C3aR in BECs, activating the microglia and impairing the NSC activities. The chemokine CCL5 released by BECs recruits the resident brain microglia. Considering the crucial roles of BECs in neurogenesis and myelination, the studies of the contents and functions of BECs secretions in the regeneration failure of neurons and oligodendrocytes can provide insights into the pathophysiological process of neurodegenerative diseases. BECs, barrier type brain endothelial cells; NSCs, neural stem cells; TGFβ, transforming growth factor beta; OPCs, oligodendrocyte precursor cells; NPCs, neural progenitor cells; SHH, sonic hedgehog; VEGF, vascular endothelial growth factors; BBB, blood–brain barrier; GM-CSF, granulocyte-macrophage colony-stimulation factor; ApoE4, apolipoprotein E4; VCAM1, vascular cell adhesion molecule 1.

Other than the well-accepted neurovascular niche, some studies raise the concept of “oligovascular niche,” indicating the close bidirectional crosstalk between oligodendrocyte precursor cells (OPCs) and BECs in white matter. Oligodendrocytes are the myelinating cells around axons of neurons in the CNS, providing metabolic support and maintaining the rapid conduction function in axons. OPCs are stem cells that persist into adulthood and generate oligodendrocytes throughout life. They regenerate the damaged myelin during the process of remyelination in demyelination diseases, such as MS. Effective remyelination by OPCs can restore metabolic support to the axon, thus limiting the axon degeneration and neurodegeneration (Setzu et al., 2004). Recent experimental evidence demonstrates that BECs receive signals from OPCs and have the potential to regulate OPCs in different states. OPCs induce white matter angiogenesis by hypoxia-inducible factor (HIF) and Wnt signaling pathway (Yuen et al., 2014; Chavali et al., 2020). On the other hand, BEC-derived TGFβ1 regulates the specification of OPCs from neural progenitor cells (NPCs) (Paredes et al., 2021). After the establishment of early BBB in the neonatal mouse brain, OPCs were found emerging and migrating along vessels. This process still exists in mice lacking pericytes but disrupts in mice lacking endothelium, indicating that interaction with the brain vascular endothelium is required by developing OPCs (Tsai et al., 2016). In certain human active MS lesions, aberrant clustering of OPCs is identified around CD31 positive vasculature, indicating a defective OPC detachment from BECs during perivascular migration (Niu et al., 2019). These perivascular OPCs cause BBB disruption and MS pathology, but the reason why they are unable to detach from BECs remains unclear. Environmental signals from the surrounding niche are of great importance for stem cell activity, and the niche is dynamic as stem cell migration. Thus, how stem cells move in and out of niches is crucial for effective repair therapies. As mentioned, OPCs are in active interactions with BECs during remyelination, and the vascular niche created by BEC signals is essential for stem cell behaviors. In this case, why OPCs cannot leave the surrounding BEC niche may be a crucial early target to interfere with the endogenous repair progress of MS. Endothelial-conditioned media promote OPC vitality and proliferation, suggesting the benefits of secretions from endothelial in OPC survival and functions as well (Arai and Lo, 2009). Further research is needed in figuring out the specific substance from endothelial cells and clarifying the contribution of it in the OPC functions, which may be a potential target in demyelinating and neurodegeneration diseases (Figure 2).

## 4. Conclusion

So far, vascular dysfunction has emerged as a common problem in the early stages of all human neurodegenerative diseases. More studies challenge the traditional opinion that BECs are indolent and passive barrier cells in BBB Recent technical advances such as single-cell sequencing have helped us notice the crucial roles of BECs in neurodegenerative diseases.

In the pathogenesis of neurodegenerative diseases, pathological proteins are crucially linked to BEC dysfunction in most neurodegenerative disorders, and damaged BEC transport accelerates the deposition of pathological proteins. Moreover, recent studies uncover more pathological-protein-independent neurodegeneration, especially neuroinflammation induced by activated BECs. The relative contributions of interactions between microglia and BECs to inflammation in neurodegenerative diseases are needed to be examined. In addition, a thorough knowledge of the link between BEC signals and disease resilience is required. Although neurogenesis failure in neurodegenerative diseases is still controversial, we ought to attend to the roles of stem cells in the process of diseases. The behaviors of OPC have emerged as an important area in the field of demyelinating diseases such as MS. Considering the regulations of BECs in the stem cell behaviors and the vascular niche created by BEC secretions, we need to understand whether BEC dysfunction is the reason for the regeneration failure of neurons. In summary, the molecular basis of BEC dysfunction will be crucial for the understanding of the pathological roles of vascular dysfunction and the development of new therapies during the early phase of neurodegenerative diseases.

## Author contributions

QD, MC, JS, and YY researched data for the review, wrote and revised the manuscript, contributed substantially to discussions of its content, and undertook review and editing of the manuscript before submission. All authors read, revised, and approved the final manuscript.
